# Hepcidin is a Better Predictor of Iron Stores in Premenopausal Women than Blood Loss or Dietary Intake

**DOI:** 10.3390/nu8090540

**Published:** 2016-09-02

**Authors:** Karen H. C. Lim, Alison O. Booth, Caryl A. Nowson, Ewa A. Szymlek-Gay, David O. Irving, Lynn J. Riddell

**Affiliations:** 1 Institute for Physical Activity and Nutrition, Melbourne Burwood Campus, Deakin University, 221 Burwood Highway, Burwood, VIC 3125, Australia; alison.booth@deakin.edu.au (A.O.B.); caryl.nowson@deakin.edu.au (C.A.N.); ewa.szymlekgay@deakin.edu.au (E.A.S.-G.); lynn.riddell@deakin.edu.au (L.J.R.); 2 Australian Red Cross Blood Service, 17 O’Riordan Street, Alexandria, New South Wales 2015, Australia; dirving@redcrossblood.org.au; 3 Faculty of Health, University of Technology Sydney, 15 Broadway, Ultimo, New South Wales 2007, Australia

**Keywords:** iron status, female, blood donation, menstrual loss, iron intake, hepcidin

## Abstract

The relationship between dietary intake, circulating hepcidin and iron status in free-living premenopausal women has not been explored. This cross-sectional study aimed to identify dietary determinants of iron stores after accounting for blood loss and to determine whether iron intake predicts iron stores independently of hepcidin in a sample of Australian women. Three hundred thirty eight women aged 18–50 years were recruited. Total intake and food sources of iron were determined via food frequency questionnaire; the magnitude of menstrual losses was estimated by self-report; and blood donation volume was quantified using blood donation records and self-reported donation frequency. Serum samples were analysed for ferritin, hepcidin and C-reactive protein concentrations. Linear regression was used to investigate associations. Accounting for blood loss, each 1 mg/day increase in dietary iron was associated with a 3% increase in iron stores (*p* = 0.027); this association was not independent of hepcidin. Hepcidin was a more influential determinant of iron stores than blood loss and dietary factors combined (*R*^2^ of model including hepcidin = 0.65; *R*^2^ of model excluding hepcidin = 0.17, *p* for difference <0.001), and increased hepcidin diminished the positive association between iron intake and iron stores. Despite not being the biggest contributor to dietary iron intake, unprocessed meat was positively associated with iron stores, and each 10% increase in consumption was associated with a 1% increase in iron stores (*p* = 0.006). No other dietary factors were associated with iron stores. Interventions that reduce hepcidin production combined with dietary strategies to increase iron intake may be important means of improving iron status in women with depleted iron stores.

## 1. Introduction

Iron deficiency is the most prevalent nutritional disorder worldwide [1]. It has been estimated that in Australia, 12% of women aged between 16–44 years present with depleted iron stores (serum ferritin < 15 µg/L [2]) and are thus more likely to experience impaired physical performance [3] and potentially impaired cognitive ability [4,5,6]. Iron homeostasis is maintained via intestinal absorption, with iron absorption increasing when iron status is impaired [7,8]. Absorption is determined in part by hepcidin, a peptide hormone produced by the liver in response to conditions including iron concentration [9]. Hepcidin regulates the export of iron into circulation by binding to the iron transport protein, ferroportin, thereby trapping iron in enterocytes and macrophages [9]. When the iron concentration of the body is low, hepcidin production is suppressed, and iron absorption and cycling increase [9].

Dietary influences of iron absorption include the form of iron and simultaneous consumption of other food components. Heme iron is more bioavailable than non-heme iron [10], and the bioavailability of iron in a whole diet is increased by the consumption of ascorbic acid and meat protein [11]. In single meals, iron absorption is reduced by consumption of phytate [12,13] and potentially calcium [13,14], as well as polyphenols in tea and coffee [15,16], although data from free-living populations are lacking. Iron intake has a positive influence on iron status, with each 1 g/day increase of elemental iron associated with a 1 µg/L increase in serum ferritin [17]. However, there exists inter-individual variation in iron absorption that may be caused by genetic variation in hepcidin expression [18,19,20]. It has been postulated that this variation in expression may be a greater influence of iron absorption than dietary factors [21], but there is no evidence exploring the relationships between dietary intake, hepcidin concentration and iron status in free-living individuals. In a single-meal study, Zimmermann et al. [20] found that circulating hepcidin explained a ”modest” 28% of variance in absorption, leading the authors to suggest that additional physiological and genetic factors are important in influencing absorption [20]. 

As iron concentration of the body influences hepcidin expression, blood loss, as a major source of iron loss and, thus, a determinant of iron concentration, should be considered when investigating determinants of iron stores. The progressively deleterious effect of regular blood donation on iron stores has been recognized since the late 1970s [22], and greater menstrual losses increase women’s risk of iron deficiency [23]. Erythropoietic activity leads to a reduction in hepcidin expression [9], and hepcidin concentrations decline with repeated blood donations, although there is a great deal of variance in this response [24]. Furthermore, in inflammatory states, ferritin and hepcidin concentrations are both elevated, and if prolonged, iron absorption can be suppressed, resulting in anaemia of inflammation [25]. Pro-inflammatory conditions may impair iron status, and there is evidence of higher circulating hepcidin concentrations and impaired iron status in women with obesity [26,27]. There is therefore a need to account for inflammation when investigating ferritin and hepcidin. As hepcidin measurement is relatively novel, there are no published studies considering the combined effect of blood loss, iron intake and hepcidin on iron status. Studies investigating predictors of iron stores in premenopausal women, including blood donation, menstrual losses or oral contraceptive use, and intakes of iron or iron-rich foods have only predicted 9% of variance in serum ferritin [28,29]. These data indicate that other factors must play a significant role in determining iron status, and one of these factors may be hepcidin. The aims of this study were to identify dietary determinants of iron stores after accounting for blood loss and to determine whether iron intake predicts iron stores independently of circulating hepcidin levels in a sample of premenopausal women.

## 2. Materials and Methods 

### 2.1. Study Design and Participants

Premenopausal women 18–50 years of age were recruited for this cross-sectional study as described elsewhere [30]. Briefly, 396 women were recruited from the student and staff population at Deakin University (Burwood, Melbourne, Australia), from blood donors registered with the Australian Red Cross Blood Service (Blood Service, Sydney, Australia) and from metropolitan Melbourne from July 2010–January 2014. All participants provided written informed consent at the time of recruitment. Ethics approval for data collection between 2010 and 2011 was granted by the Deakin University Human Research Ethics Committee (Reference 2009-191) and by the Australian Red Cross Blood Service Ethics Committee (Reference 2010#01). Ethics approval for data collection between 2012 and 2014 was granted by the Deakin University Human Research Ethics Committee (Reference 2012-046).

**Figure 1:**
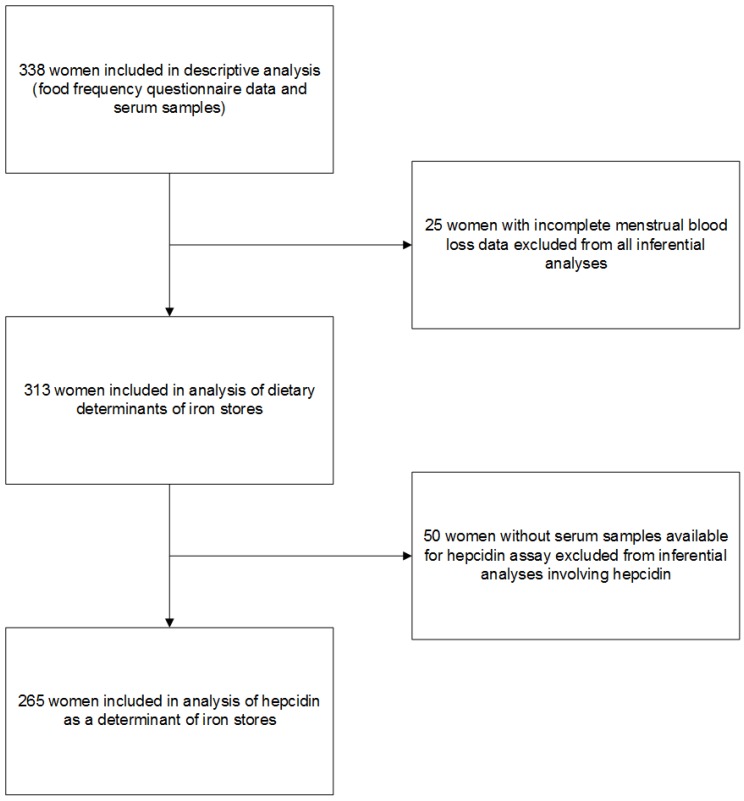
Study flow diagram and inclusion of women in analyses.

Women were eligible to participate if they had not been through menopause and if they were not currently pregnant or lactating and had not been in the previous 6 months. The presence of chronic disease was not an exclusion criterion. Participants were asked to complete a demographic and health background questionnaire, a food frequency questionnaire (FFQ) and to provide a blood sample. Of the 382 women who completed both questionnaires, 342 also provided a blood sample. Data from four women were excluded from analyses: three women reported energy intakes > 3 SD from the mean (>14,846 kJ/day), and one woman had recently received an intravenous iron infusion (serum ferritin = 461 µg/L). In total, 338 women were included in the present study, 313 in the analysis of dietary determinants of iron stores and 265 in the analysis of hepcidin as a determinant of iron stores (Figure 1).

### 2.2. Assessment of Usual Dietary Intake

Women’s usual dietary intakes over the past 12 months were recorded using the Cancer Council Victoria Dietary Questionnaire for Epidemiological Studies v3.1 (DQES v3.1), a paper-based FFQ that assesses the consumption frequency of 140 foods and beverages and can be used to estimate nutrient intakes [31]. Dietary iron intake reported in the previous version of this FFQ [31] has been validated against seven-day weighed food records [32]. As explained previously [30], nutritional analysis of DQES v3.1 was conducted independently of the Cancer Council Victoria. Usual nutrient intakes per day, including mg/day dietary iron and ascorbic acid, were calculated using Australian food composition databases NUTTAB 2010 [33] and AUSNUT 2007 [34] via FoodWorks 7 (Xyris Software, Queensland, Australia) [30]. As the Australian food composition databases do not include phytate, daily phytate intake was estimated separately using the University of Otago’s (Dunedin, New Zealand) database of the phytate content of 5775 foods and beverages [35]. This database was based on published literature of phytate concentration in foods, which were adjusted to reflect food processing methods in New Zealand [35], and was considered by the authors as the most appropriate for an Australian population. Phytate intakes were computed using Microsoft Excel 2013 (Microsoft Corporation, Redmond, WA, USA). 

Food group contributions to dietary iron intakes were estimated following the food grouping system used in the 2011 Australian Health Survey (AHS 2011) [36]. As with AHS 2011 [37], these contributions were estimated at the group level: the mean mg/day contributed by each AHS 2011 major and sub-major food group was divided by the mean mg/day dietary iron intake of the cohort and multiplied by 100 to present as a percentage.

### 2.3. Assessment of Supplemental Iron Intake

Use of dietary supplements containing iron was self-reported. Women were asked to write the name of any supplements they were currently using that contained iron, the frequency of consumption and the dose. The dose of supplemental iron (as elemental iron) was confirmed by checking supplement packaging or the manufacturers’ websites. To estimate supplemental iron intake per day, the total amount of iron from all supplements consumed per week was divided by seven days. Supplemental iron intakes were added to dietary intakes to determine total iron intakes per day. 

### 2.4. Assessment of Blood Loss

The sources of blood loss included in this study were blood donation, menstrual losses and nosebleeds. 

The blood donation history of women recruited at the Blood Service was sourced from Blood Service records. The information consisted of the number of donations made over the past 12 months and the donation type at each visit (whole blood or apheresis). The volume of blood donated over the past year was quantified using conversion factors provided by the Blood Service. Each whole blood donation was considered to be 470 mL, and each apheresis (plasmapheresis and plateletpheresis) donation was considered to be 60 mL of whole blood. 

Women recruited at Deakin University in 2010 self-reported their blood donor status and frequency of donation. This information was converted to mL of blood donated over the past year using the factors noted above. Women recruited at Deakin University in 2012–2014 had not donated blood in the past two years as an eligibility criterion.

A previously-published questionnaire was used to record self-reported menstrual blood loss [38]. Women were asked to report menstrual characteristics, including frequency of menses per year; the number of heavy and light days during menses; and the number, type and brand of sanitary pads and tampons used on heavy and light days. This information was used to calculate an arbitrary menstrual blood loss score for each woman per menses and per year. This questionnaire assessing menstrual blood loss per menses has been validated among women aged 18–29 years in New Zealand where the arbitrary menstrual blood loss score was positively and moderately correlated with the weight of women’s menstrual products [38]. 

Experience of nose bleeds was recorded using a categorical question (i.e., “Do you get nose bleeds?” (Yes/No)).

### 2.5. Biochemical Analyses

Participants were asked to provide blood samples that were analysed for serum ferritin, haemoglobin, C-reactive protein (CRP) and hepcidin concentrations. Women recruited in Melbourne were asked to provide a fasting intravenous blood sample that was collected and analysed for serum ferritin, haemoglobin and CRP by Dorevitch Pathology (Heidelberg, Victoria, Australia), a commercial pathology laboratory. Serum ferritin was measured using the ADVIA Centaur Ferritin Assay on the Siemens ADVIA Centaur (Siemens Healthcare Diagnostics, Deerfield, IL, USA), haemoglobin measured using the Sysmex Automated Hematology Analyzer XE-2100 (Sysmex, Kobe, Japan) and CRP measured using a latex-enhanced immunoturbidimetric assay on a Siemens ADVIA 2400 (Bayer Diagnostics, Tarrytown, NY, USA). Women recruited at the Blood Service provided non-fasting blood samples as sampling occurred just prior to a blood donation, and the Blood Service performed analysis of serum ferritin, haemoglobin and CRP. At the Blood Service, intravenous samples were used for serum ferritin and CRP analyses and capillary finger-stick samples for haemoglobin analysis. Serum ferritin was measured using the AxSYM Ferritin assay on the AxSYM (Abbott Diagnostics, Abbott Park, IL, USA); haemoglobin was measured using the Hemocue B-Hemoglobin Photometer (Hemocue, Angelholm, Sweden); and CRP was measured using the Quantikine Human CRP Immunoassay (R & D Systems, Minneapolis, MN, USA). The time of blood sampling was recorded by staff at Dorevitch Pathology and the Blood Service. Blood sampling took place between 7:40 and 18:30 and 8:00 and 16:55 for the Melbourne and Sydney cohorts, respectively. Both Dorevitch Pathology and the Blood Service are accredited with the National Association of Testing Authorities (Australia) in conjunction with the Royal College of Pathologists of Australasia and comply with the requirements of ISO 15189:2012. For all participants, at the time of sampling, an aliquot of serum was stored at −70 °C at Deakin University (Burwood, Victoria, Australia) for hepcidin analysis. Serum hepcidin was analysed at Deakin University using a competitive enzyme immunoassay kit for hepcidin-25 (catalogue number S-1337, Bachem/Peninsula Laboratories, San Carlos, CA, USA). 

### 2.6. Assessment of Covariates

The demographic and health background questionnaire given to participants collected information including country of birth, highest education, employment status, smoking status and use of oral contraception. All participants had weight measured. Women from Melbourne had height measured using a wall-mounted stadiometer, while women from Sydney self-reported height as part of the background questionnaire. Body mass index (BMI) was calculated as weight (kg) divided by height (m^2^).

### 2.7. Statistical Analysis

All statistical tests were two-sided, and statistical significance was defined as *p* < 0.05. Analyses were conducted using Stata/SE 13.1 (StataCorp, College Station, TX, USA). Descriptive statistics are presented as *n* (%) or the mean (95% CI). The normality of variables was assessed through visual inspection of histograms, and data were natural log-transformed and presented as the geometric mean (95% CI) if the distribution was not normal. Normality was confirmed after log transformation.

For descriptive statistics, serum ferritin concentrations were multiplied by a factor of 0.65 if CRP > 5 mg/L (*n* = 34) to correct serum ferritin concentrations for inflammation [39]. Inferential statistics used uncorrected serum ferritin values with CRP included as a covariate in models. As hepcidin concentrations are also elevated in an inflammatory state [25], inclusion of CRP in inferential analyses was required. Women were categorized as having low iron stores if inflammation-corrected serum ferritin values < 15 µg/L and haemoglobin values ≥ 120 g/L and categorized as having iron-deficiency anaemia if serum ferritin values < 15 µg/L and haemoglobin values < 120 g/L [40].

The inferential analysis was performed in three steps. Firstly, blood loss, demographic and anthropometric characteristics outlined in Section 2.6 were selected in a linear regression model predicting serum ferritin using automated backwards selection with the criterion of *p* ≤ 0.2 [41]. Dietary characteristics selected a priori were then added to the model to test: (a) intakes of the major food sources of iron and an absorption inhibitor (phytate) and enhancer (ascorbic acid); (b) dietary intakes of iron, phytate and ascorbic acid; and (c) total intakes of iron (dietary + supplemental iron) and dietary intakes of phytate and ascorbic acid. To explore whether an inhibitory effect of phytate on an association between iron intake and iron stores is dependent on ascorbic acid intake, we included an interaction between mg/day intakes of phytate, iron and ascorbic acid. Secondly, we included hepcidin concentration in multivariate models of serum ferritin that included dietary or total iron intake. To further investigate the impact of hepcidin, we included an interaction between iron intake (mg/day) and serum hepcidin (ng/mL) on serum ferritin concentrations, and this interaction was visualized using the post-estimation “margins” command. The time of blood sampling was included as a covariate in models with hepcidin to account for diurnal variation [42]. Natural log-transformation of serum ferritin, CRP, hepcidin, meat consumption and phytate intake was used to correct skewness that violated assumptions of regression residuals. The presence of collinearity among independent variables was determined using the criterion of *r* ≥ 0.8 [41], with Pearson’s correlation used for normally-distributed variables and Spearman’s correlation used for skewed variables.

## 3. Results

Women in this study were aged on average 29 (95% CI 28, 30) years, and most were in the healthy weight range (Table 1). In the study sample, the prevalence of low iron stores (using serum ferritin values corrected for inflammation in 34 women) in the absence of anaemia was 30% (*n* = 100), and an additional 7% (*n* = 22) presented with iron-deficiency anaemia. 

One-third of dietary iron consumed by the study sample was sourced from cereals and cereal products (including breakfast cereals and breads); 16% was sourced from vegetable products and dishes; and 14% was sourced from meat and poultry (Table 2).

### 3.1. Predictors of Iron Stores

Of the blood loss, demographic and anthropometric characteristics, only blood losses through blood donation and menstruation met the criterion for selection into the model. Although BMI and CRP were moderately and positively correlated (Spearman’s *r* = 0.35, *p* < 0.001), BMI was not selected into the model as a predictor of serum ferritin. Age, BMI, education, employment, status as a smoker and having had children were not associated with serum ferritin. Including a dichotomous variable of location (Sydney/Melbourne) did not change the associations between blood loss and serum ferritin. When the dietary characteristics were added to the model, the multivariate analyses (Table 3) demonstrated that usual dietary iron intake was a determinant of iron stores, with each 1 mg/day increase in dietary iron associated with a 5% (95% CI 1, 8%) increase in serum ferritin and each 10% increase in total iron (dietary + supplemental iron) intake associated with a 3% (95% CI 1, 5%) increase in serum ferritin. Applying this information to the study sample, a 1 mg/day increase in dietary iron or a 10% increase in total iron intake would see the serum ferritin geometric mean increase from approximately 20–21 µg/L. Dietary sources of iron were not equally associated with serum ferritin. Intakes of cereals and cereal products (including breakfast cereals and breads) and vegetable products and dishes were not associated with iron stores (β for cereals = 0.090, 95% CI −0.080, 0.260; β for vegetables = 0.001, 95% CI 0, 0.002), despite contributing 30 and 16% of dietary iron intake, respectively. However, a small positive association was observed between iron stores and total unprocessed meat consumption (β for total meat = 0.089, 95% CI 0.006, 0.171), which contributed to 14% of dietary iron intakes. Each 10% increase in intakes of combined red and white meat corresponded to a 0.9% (95% CI 0.6, 1.7%) increase in serum ferritin (e.g., an increase in total meat consumption from 78–86 g/day would equate to serum ferritin increasing from 20.1–20.3 µg/L). Usual dietary intakes of ascorbic acid and phytate were not associated with iron stores, and a three-way interaction between intakes of phytate, ascorbic acid and iron was not significant (data not shown). For each 100 mL of whole blood donated, serum ferritin decreased by 8% (95% CI 6, 10%), and for each arbitrary unit increase in menstrual loss, serum ferritin decreased by 0.2% (95% CI 0.1, 0.4%).

### 3.2. Association between Iron Intake and Iron Stores Independent of Hepcidin

**Figure 2:**
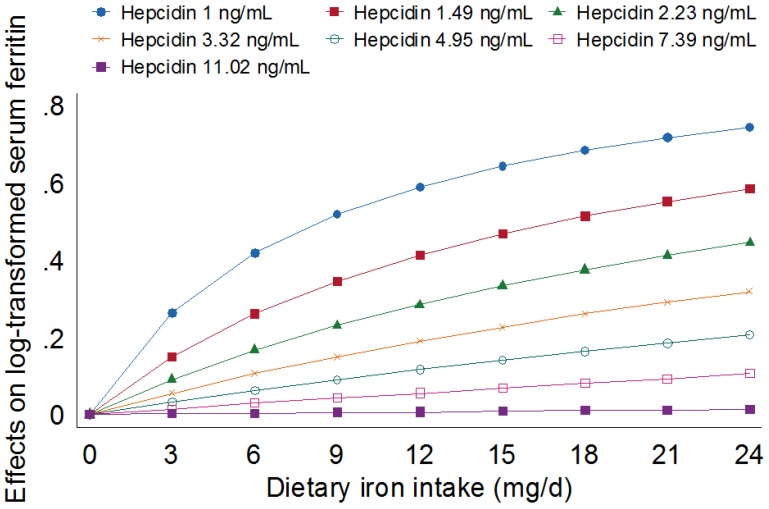
Effect of serum hepcidin on the relationship between dietary iron intake and natural log-transformed serum ferritin concentration, holding CRP, blood donation and menstrual losses at their mean values (CRP 0.67 mg/L, blood donation 400 mL/year, menstrual loss 104 arbitrary units, blood sampling at 11:15). The association between dietary iron intake and natural log-transformed serum ferritin is non-significant at 7.39 and 11.02 ng/mL (hepcidin concentrations > 11.02 ng/mL not depicted).

Hepcidin data were available for 265 women. When the multivariate analyses were repeated in only these 265 women, no differences were observed in outcomes compared with the analyses that included 313 women (data not shown). There was a positive correlation between hepcidin and serum ferritin, with Spearman’s *r* = 0.67, *p* < 0.001. Including natural log-transformed hepcidin concentration in models increased the amount of explained variance in serum ferritin from 20% (Table 3) to 65% (Table 4). The relationship between log-transformed serum ferritin and hepcidin also remained when repeating the analysis (Table 4, Model A) in only the women who provided fasting morning samples (β for log-transformed serum hepcidin 0.86 (95% CI 0.72, 1.01)). Furthermore, dietary iron intake was no longer associated with iron stores when hepcidin was included as a covariate, although the positive association with total iron intake remained. We also explored interactions between dietary iron intake or total iron intake and circulating hepcidin in these models. Including an interaction of dietary iron intake (mg/day) × log-transformed hepcidin was found to be negative and statistically significant (β-0.030 (95% CI −0.053, −0.007)) and indicated that a positive association between dietary iron intake and iron stores was strongest when circulating hepcidin concentrations were low. This association remained when a variable indicating the location of recruitment (Sydney/Melbourne) was added to the model. Figure 2 displays this interaction at different levels of hepcidin concentration. The change in the slope of natural log-transformed serum ferritin on dietary iron intakes was predicted across the sample’s range of natural log-transformed hepcidin values from 0–3.2 (1–24.54 ng/mL) at intervals of 0.4, holding blood loss covariates at their mean. The positive association between dietary iron intake and log-transformed serum ferritin was strongest at 1 ng/mL hepcidin (i.e., greatest changes in slope), progressively weakening as the intervals of hepcidin concentrations increased. At 7.39, 11.02, 16.44 and 24.53 ng/mL hepcidin, there was no association between dietary iron intake and iron stores.

## 4. Discussion

Although dietary iron intake was associated with iron stores in this sample of premenopausal women, intake did not predict iron stores independently of circulating hepcidin concentration. Circulating hepcidin was notably more influential and explained more variance in iron stores than diet and blood loss combined (65% of variance including hepcidin, 20% of variance, excluding hepcidin). Furthermore, this study demonstrated that increasing concentrations of circulating hepcidin suppressed the association between usual dietary iron intake and iron stores, indicating the physiological significance of hepcidin in maintaining iron stores in free-living women. The relationship between dietary intake and iron stores was strongest at the lowest levels of circulating hepcidin, and as hepcidin concentration increased, the association between iron stores and intake weakened, such that there was no relationship when hepcidin was greater than 7.39 ng/mL. This value is presented tentatively, as it has been noted that absolute hepcidin values differ across measurement methods, likely due to the hepcidin values manufacturers assign to assay standards [44]. However, these results are in line with the work demonstrating that the amount of iron absorbed is inversely associated with circulating hepcidin [18,19,20] and also demonstrate the influence of hepcidin in the determination of iron stores. These findings are important, as they demonstrate the necessity for an increased understanding of determinants of hepcidin and the potential to influence hepcidin concentrations. While genetic factors have a significant influence on circulating concentrations of hepcidin (for a review see [45]), identifying determinants that can be modified through lifestyle changes or other interventions may help prevent or treat iron deficiency related to increased hepcidin production. Recent work has shown that black soybean extract can inhibit hepcidin production and increase circulating iron in animal models [46], indicating that hepcidin can be actively modified, and the expression is not only a response to genetic influences, inflammation or hypoxic states. Interestingly, we did not observe a significant interaction between total iron intake (i.e., dietary and supplemental iron) and hepcidin in this sample of women. As only 20% of the sample reported taking supplements containing iron, the study may have been underpowered to detect any influence of supplements. It is also possible that more information on women’s supplemental use, such as duration of use, was required. Supplement users may have included women who had only been taking iron supplements for a short period, before the supplemental iron may have been reflected in iron stores.

In our sample of women, each 1 mg/day increase in dietary iron intake corresponded to a 5% increase in serum ferritin. Similar associations were observed when iron from supplements was included, such that a 10% increase in total iron intake corresponded to a 3% increase in iron stores. These quantifications of the influence of iron intake on iron stores are unique, as most cross-sectional work in premenopausal women has not found positive associations between dietary iron intake and iron status [47]. We also observed that the source of dietary iron appeared to influence iron stores. Although dietary iron intake was a significant determinant of iron stores, intakes of the two major food groups contributing to dietary iron intake were not: cereals (contributing 31% of dietary iron) and vegetables (contributing 16%). In contrast, consumption of meat (beef, lamb, chicken, pork; combined contribution 14%) was consistently positively associated with iron stores whether considered separately as red or white meat or combined. This finding adds to the body of existing evidence supporting the positive effect of meat consumption on iron absorption and iron status [11,29,48,49,50], which may be due to the more efficient absorption of heme iron compared to non-heme iron [51]. The relationship may also represent the overall diet quality of women consuming unprocessed meats, rather than meat intake itself. For example, Beck et al. used factor analysis to describe dietary patterns among a cohort of premenopausal women and found that women adhering to a pattern characterized by high intakes of beef and poultry (distinct from “prepared meats”), as well as broccoli, carrots and capsicum were 41% less likely to present with serum ferritin < 20 µg/L [52]. We did not observe any associations between consumption of iron absorption enhancers other than meat (e.g., ascorbic acid) or potential inhibitors (e.g., phytate, calcium, tea and coffee tannins) and iron stores, an unsurprising finding, given that we did not account for the timing of consumption. Timing of consumption may be an important influence, and it has been demonstrated that beverage tannins have no effect on iron absorption if consumed one hour prior to food, but hinder iron absorption if consumed with or one hour following food [16]. Therefore, further research that can account for women’s habitual meal patterns is required.

Although there was a positive, moderate correlation between CRP and BMI, there was no association between serum ferritin and BMI in our sample of women. Previous studies have reported inverse relationships between iron status and BMI [27], and the lack of a relationship in the current study may be due to the small proportion of participants with BMI in the obese range (9%). Further, lower iron status has been observed in women with obesity compared to women of normal weight [26], but mean BMI in these obese women (50 kg/m^2^) was much higher than in the present study. As the prevalence of obesity in Australia has steadily increased over time [53], there is a need for work that can ascertain whether and at what stage excess adiposity impairs iron status. 

The outcomes from our study need to be considered in light of the following limitations. First, our study sample of women was a self-selected group that was not representative of premenopausal women in Australia. Women who participated in this study were more likely to fall in the healthy weight range [54], less likely to smoke [54] and more likely to be tertiary educated [55] compared to the general population. While a lack of representativeness limits confident extrapolation of prevalence data and point estimates, we were still able to identify factors associated with impaired iron stores and an effect of hepcidin in line with current understanding. Although, as with all cross-sectional studies, causal pathways could not be determined in this research, directionality could be inferred from the assessed determinants. For example, negative associations between blood loss and iron stores implied increased blood loss reduced iron stores, rather than reduced iron stores increasing blood loss. Nonetheless, stronger study designs are needed to substantiate the dietary determinants assessed in this research. We also acknowledge that immunoassays for hepcidin-25 can demonstrate cross-reactivity with hepcidin-20 and hepcidin-22 [56], isoforms that do not affect iron metabolism, and research with more specific measurement of hepcidin-25 is needed.

## 5. Conclusions

In conclusion, we observed circulating hepcidin to be a major determinant of iron stores, and increased levels of hepcidin suppressed the association between dietary iron intake and iron stores in free-living premenopausal women. We also found meat consumption to be a positive determinant of iron stores despite cereals and vegetables contributing more dietary iron. These findings support the importance of unprocessed meat as part of a balanced diet for premenopausal women and indicate that further research should investigate the potential for lifestyle interventions to regulate hepcidin expression.

## Figures and Tables

**Table 1:** Characteristics of the study sample of premenopausal women aged 18–50 years ^a^.

	Total (*n* = 338)
**Demography and anthropometry**	
Age (years)	29.1 (28.3, 29.9)
Body mass index (kg/m^2^)	24.0 (23.6, 24.4)
Underweight ^b^	11 (3%)
Normal weight ^b^	224 (67%)
Overweight ^b^	69 (21%)
Obese ^b^	29 (9%)
Tertiary education	274 (81%)
Currently employed	297 (89%)
Current smoker	26 (7%)
Has children	55 (16%)
**Diet and supplements**	
Dietary iron intake (mg/day)	10.4 (10.0, 10.8)
Taking supplements containing iron	71 (21%)
Supplemental iron intake among users (mg/day, *n* = 71) ^c^	7.2 (5.3, 9.8)
Total iron intake (diet + supplements) ^c^	11.4 (10.7, 12.0)
Dietary ascorbic acid intake (mg/day)	121 (115, 127)
Phytate intake (mg/day)	691 (653, 733)
Energy intake (MJ/day)	7.7 (7.4, 7.9)
**Blood loss**	
Donated blood in previous 12 months	159 (47%)
Amount donated (mL/year, *n* = 159)	844 (769, 919)
Using oral contraception	125 (37%)
Menstrual blood loss units per menses ^c^	30 (28, 33)
Menstrual blood loss units per year ^c^	82 (76, 89)
Experiences nosebleeds	36 (11%)
**Biochemistry**	
Serum ferritin (µg/L) ^a,d^	19.1 (17.3, 21.0)
Venous haemoglobin (g/L, *n* = 147)	135 (133, 136)
Capillary haemoglobin (g/L, *n* = 191)	130 (129, 132)
Hepcidin (ng/mL) ^c^	5.84 (5.01, 6.79)

^a^ Values are the arithmetic mean (95% CI) unless indicated as the geometric mean (95% CI) or *n* (%). Characteristics of the 265 women with hepcidin data are nearly identical and, thus, not presented separately. ^b^ Categories of body mass index: underweight <18.50 kg/m^2^; normal range 18.50–24.99 kg/m^2^; overweight 25.00–29.99 kg/m^2^; obese >30.00 kg/m^2^ [43]. ^c^ Values are the geometric mean (95% CI). ^d^ Serum ferritin corrected for acute infection: in women with CRP ≥ 5 mg/L (*n* = 34), serum ferritin was multiplied by a factor of 0.65, as suggested by Thurnham et al. [39].

**Table 2:** Contribution of foods to dietary iron intake among the study group of women aged 18–50 years ^a,b^.

Food Group	Contribution to Dietary Iron Intake (% (95% CI))
Cereals and cereal products	30.1 (28.8, 31.5)
Breakfast cereals, ready to eat	11.9 (10.5, 13.3)
Regular breads and bread rolls (plain/unfilled/untopped varieties)	10.8 (10.1, 11.6)
Flours and other cereal grains and starches	3.7 (3.2, 4.1)
Breakfast cereals, hot porridge style	2.1(1.7, 2.5)
Pasta and pasta products (without sauce)	1.6 (1.5, 1.7)
Cereal based products and dishes	6.1 (5.7, 6.5)
Savoury biscuits	2.0 (1.7, 2.2)
Cakes, muffins, scones, cake-type desserts	1.4 (1.3, 1.6)
Pastries	1.1 (1.0, 1.3)
Vegetable products and dishes	16.1 (15.2, 16.9)
Leaf and stalk vegetables	6.2 (5.7, 6.7)
Other fruiting vegetables	2.5 (2.4, 2.7)
Cabbage, cauliflower and similar brassica vegetables	2.3 (2.1, 2.6)
Peas and beans	1.8 (1.6, 2.0)
Tomato and tomato products	1.0 (1.0, 1.1)
Carrot and similar root vegetables	1.0 (0.9, 1.1)
Meat, poultry and game products and dishes	13.9 (12.8, 15)
Beef, sheep and pork, unprocessed	8.7 (7.9, 9.5)
Poultry and feathered game	2.7 (2.4, 2.9)
Processed meat	1.9 (1.6, 2.2)
Fruit products and dishes	8.8 (8.3, 9.3)
Tropical and subtropical fruit	1.9 (1.7, 2.0)
Berry fruit	1.9 (1.7, 2.0)
Other fruit	1.8 (1.7, 2.0)
Stone fruit	1.2 (1.0, 1.3)
Legume and pulse products and dishes	3.4 (2.9, 3.9)
Seed and nut products and dishes	3.2 (2.9, 3.6)
Non-alcoholic beverages	3.2 (3.0, 3.5)
Confectionery and cereal/nut/fruit/seed bars	2.6 (2.3, 2.9)
Chocolate and chocolate-based confectionary	2.5 (2.2, 2.7)
Milk products and dishes	2.2 (2.0, 2.3)
Miscellaneous	2.1 (1.8, 2.4)
Egg products and dishes	1.9 (1.7, 2.1)
Fish and seafood products and dishes	1.7 (1.5, 1.9)
Dairy and meat substitutes	1.6 (1.2, 2.0)
Alcoholic beverages	1.3 (1.1, 1.5)
Savoury sauces and condiments	1.0 (0.9, 1.2)

^a^ Major and sub-major groups as defined in the 2011 Australian Health Survey [37]. ^b^ Major and sub-major food groups contributing < 1% of dietary iron were omitted from this list. Major groups omitted for this reason were snack foods, sugar products and dishes and fats and oils.

**Table 3:** Multivariate models of determinants of serum ferritin concentration in women aged 18–50 years (*n* = 313) ^a,b^.

	β	95% CI	*p*
Model A: Major food sources of dietary iron and intakes of dietary ascorbic acid and phytate Adj. *R*^2^ = 0.20, *p* < 0.001			
Blood donation (100 mL/year)	−0.077	−0.095, −0.059	<0.001
Arbitrary menstrual blood loss units per year	−0.002	−0.004, −0.001	0.001
Red + white meat (g/day) ^b,c^	0.089	0.006, 0.171	0.035
Cereals and cereal products (g/day) ^b,d,e^	0.090	−0.080, 0.260	0.30
Vegetable products and dishes (g/day) ^d^	0.001	0, 0.002	0.07
Ascorbic acid (mg/day)	−0.001	−0.003, 0.001	0.29
Phytate (mg/day) ^a^	−0.011	−0.193, 0.172	0.91
Model B: Dietary intakes of iron, ascorbic acid and phytate Adj. *R*^2^ = 0.20, *p* < 0.001			
Blood donation (100 mL/year)	−0.075	−0.093, −0.057	<0.001
Arbitrary menstrual blood loss units per year	−0.003	−0.004, −0.001	<0.001
Dietary iron intake (mg/day)	0.047	0.011, 0.083	0.011
Ascorbic acid (mg/day)	−0.001	−0.003, 0.001	0.23
Phytate (mg/day) ^b^	−0.094	−0.303, 0.115	0.38
Model C: Total iron intake and dietary intakes of ascorbic acid and phytate Adj. *R*^2^ = 0.21, *p* < 0.001			
Blood donation (100 mL/year)	−0.076	−0.095, −0.058	<0.001
Arbitrary menstrual blood loss units per year	−0.002	−0.004, −0.001	0.001
Total iron intake (incl. Supplements, mg/day) ^b^	0.299	0.091, 0.506	0.005
Ascorbic acid (mg/day)	−0.001	−0.003, 0.001	0.30
Phytate (mg/day) ^b^	−0.046	−0.232, 0.140	0.63

^a^ All models adjusted for C-reactive protein. ^b^ Natural log-transformed independent variable. ^c^ Red and white meat includes unprocessed beef, lamb, pork, chicken and fish. ^d^ Australian Health Survey major food group. ^e^ Cereals and cereal products includes breakfast cereals, bread, pasta and rice.

**Table 4:** Multivariate associations between dietary iron intake, total iron intake, hepcidin and natural log-transformed serum ferritin in women aged 18–50 years (*n* = 265) ^a^.

	β	95% CI	*p*
Model A: Dietary iron intake Adj. *R*^2^ = 0.65, *p* < 0.001			
Blood donation (100 mL/year)	−0.029	−0.043, −0.015	<0.001
Arbitrary menstrual loss units per year	−0.001	−0.002, 0	0.036
Dietary iron intake (mg/day)	0.011	−0.009, 0.031	0.27
Timing of sampling (h)	−0.080	−0.113, −0.047	<0.001
Hepcidin (ng/mL) ^b^	0.788	0.704, 0.872	<0.001
Model B: Total iron intake Adj. *R*^2^ = 0.66, *p* < 0.001			
Blood donation (100 mL/year)	−0.030	−0.045, −0.016	<0.001
Arbitrary menstrual loss units per year	−0.001	−0.002, 0	0.05
Total iron intake (mg/day) ^b^	0.155	0.028, 0.281	0.017
Timing of sampling (h)	−0.075	−0.109, −0.042	<0.001
Hepcidin (ng/mL) ^b^	0.784	0.699, 0.868	<0.001

^a^ All models adjusted for natural log-transformed C-reactive protein. ^b^ Natural log-transformed independent variable.
